# Expanding the spectrum of Sideroblastic Anemia with B-cell Immunodeficiency, Periodic Fever and Developmental Delay (SIFD) syndrome: a case report with new clinical insights and novel genetic variant

**DOI:** 10.3389/fimmu.2026.1876025

**Published:** 2026-08-19

**Authors:** Francesco Delle Cave, Ivan Taietti, Annalisa Agostini, Francesca Compagno, Fabio Sirchia, Grazia Bossi, Daniela Montagna, Amelia Licari, Patrizia Comoli, Tommaso Mina, Riccardo Castagnoli, Marco Zecca

**Affiliations:** 1 Pediatric Hematology/Oncology, Fondazione IRCCS Policlinico San Matteo, Pavia, Italy; 2 Pediatric Unit, Department of Clinical, Surgical, Diagnostic, and Pediatric Sciences, University of Pavia, Pavia, Italy; 3 Pediatric Clinic, Fondazione IRCCS Policlinico San Matteo, Pavia, Italy; 4 Department of Molecular Medicine, University of Pavia, Pavia, Italy; 5 Medical Genetics Unit, Fondazione IRCCS Policlinico San Matteo, Pavia, Italy; 6 Cell Factory, Fondazione IRCCS Policlinico San Matteo, Pavia, Italy

**Keywords:** anemia, immune dysregulation, inborn errors of immunity (IEI), primary immunodeficiencies (PID), sideroblastic anemia with B-cell immunodeficiency, periodic fever and developmental delay syndrome (SIFD), TRNT1, precision medicine

## Abstract

Sideroblastic Anemia with B-cell Immunodeficiency, Periodic Fever and Developmental Delay (SIFD) is a rare autosomal recessive disorder caused by biallelic pathogenic variants in the *TRNT1* gene, encoding tRNA nucleotidyltransferase 1, an enzyme essential for mitochondrial and cytosolic protein translation. The disease is characterized by a wide phenotypic spectrum, ranging from isolated hematological involvement to multisystem inflammatory disease. Herein, we report a pediatric patient with early-onset sideroblastic anemia, recurrent inflammatory episodes, B-cell immunodeficiency, immune dysregulation (cytopenia refractory to conventional treatment, splenomegaly and chronic interstitial lung disease), ectodermal dysplasia, mild developmental delay with white-matter hypomyelination, and progressive multisystem involvement. Genetic testing identified two biallelic *TRNT1* variants, the novel c.841G>C variant and the previously reported pathogenic c.1252dupA variant, inherited from the father and mother, respectively, confirming the diagnosis of SIFD. The patient required immunoglobulin replacement therapy and immunomodulatory treatment, including rituximab, with partial control of infectious and immune dysregulation manifestations. This case expands the clinical, immunological, and genetic spectrum of TRNT1-related disease and highlights that immune dysregulation may dominate the clinical course. SIFD should be considered in children presenting with sideroblastic anemia associated with recurrent unexplained inflammation, B-cell immunodeficiency, cytopenias, and multisystem involvement. Early genetic diagnosis is essential to guide monitoring, therapeutic decisions, and prognostic stratification.

## 1 Introduction

Sideroblastic anemia with B-cell immunodeficiency, periodic fever, and developmental delay (SIFD) is a rare autosomal recessive disorder, first described in (1), caused by biallelic pathogenic variants in the TRNT1 gene, which encodes tRNA nucleotidyltransferase 1 (2). This enzyme catalyzes the addition of the CCA trinucleotide to the 3’ end of both cytosolic and mitochondrial tRNAs, a step essential for aminoacylation and protein synthesis (3). Defective TRNT1 function leads to impaired tRNA maturation, mitochondrial dysfunction, cellular stress, and activation of inflammatory pathways, thereby contributing to the hematological, immunological, neurological, and systemic manifestations observed in affected patients (4). Although the classical phenotype includes sideroblastic anemia, periodic fever, immunodeficiency, and developmental delay, increasing evidence indicates that SIFD is characterized by a broad clinical spectrum, with variable severity and heterogeneous organ involvement (5). Maccora et al. recently provided a systematic review of the phenotypic spectrum and genetic variants reported in the literature, highlighting the marked clinical heterogeneity of this condition and the lack of standardized therapeutic guidelines (6). Since then, additional cases have further expanded the clinical and genetic landscape of SIFD, with 73 cases carrying *TRNT1* variants reported worldwide to date (7–14). Although immune dysregulation is increasingly recognized as a key feature of SIFD, the full extent of immunological involvement remains incompletely understood. Here, we report a pediatric patient with SIFD carrying a novel *TRNT1* variant, presenting with progressive B-cell immunodeficiency, recurrent inflammation, severe immune dysregulation, cytopenias, splenomegaly, and multisystem involvement. This case further expands the clinical, immunological, and genetic spectrum of TRNT1-related disease.

## 2 Case description

**Figure 1:**
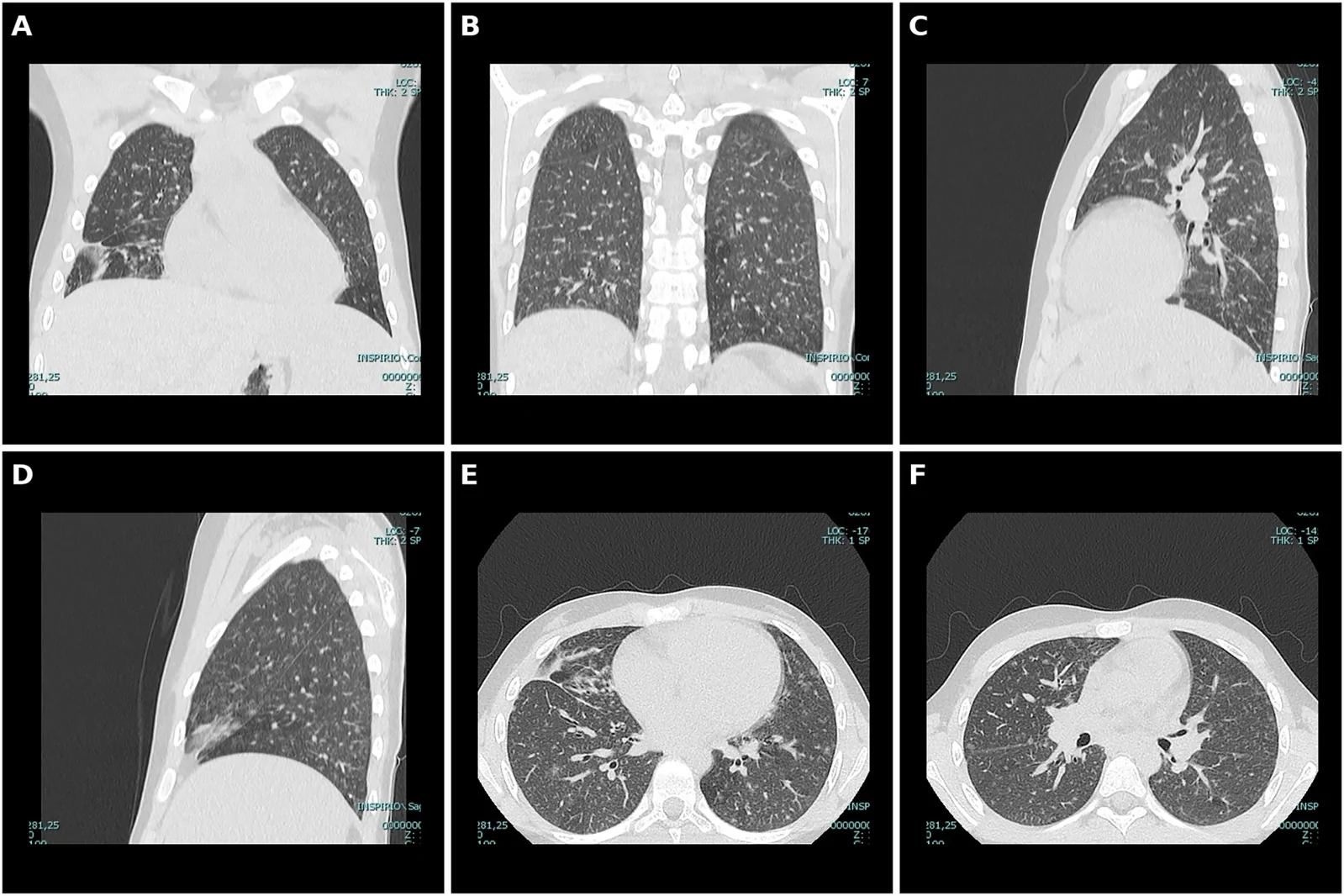
Chest computed tomography findings. **(A–B)** Coronal CT scan showing bilateral diffuse interstitial involvement. **(C–D)** Sagittal CT scan highlighting peribronchovascular thickening and patchy ground-glass opacities. **(E–F)** Axial CT scan showing subpleural consolidations and ground-glass opacities.

**Figure 2:**
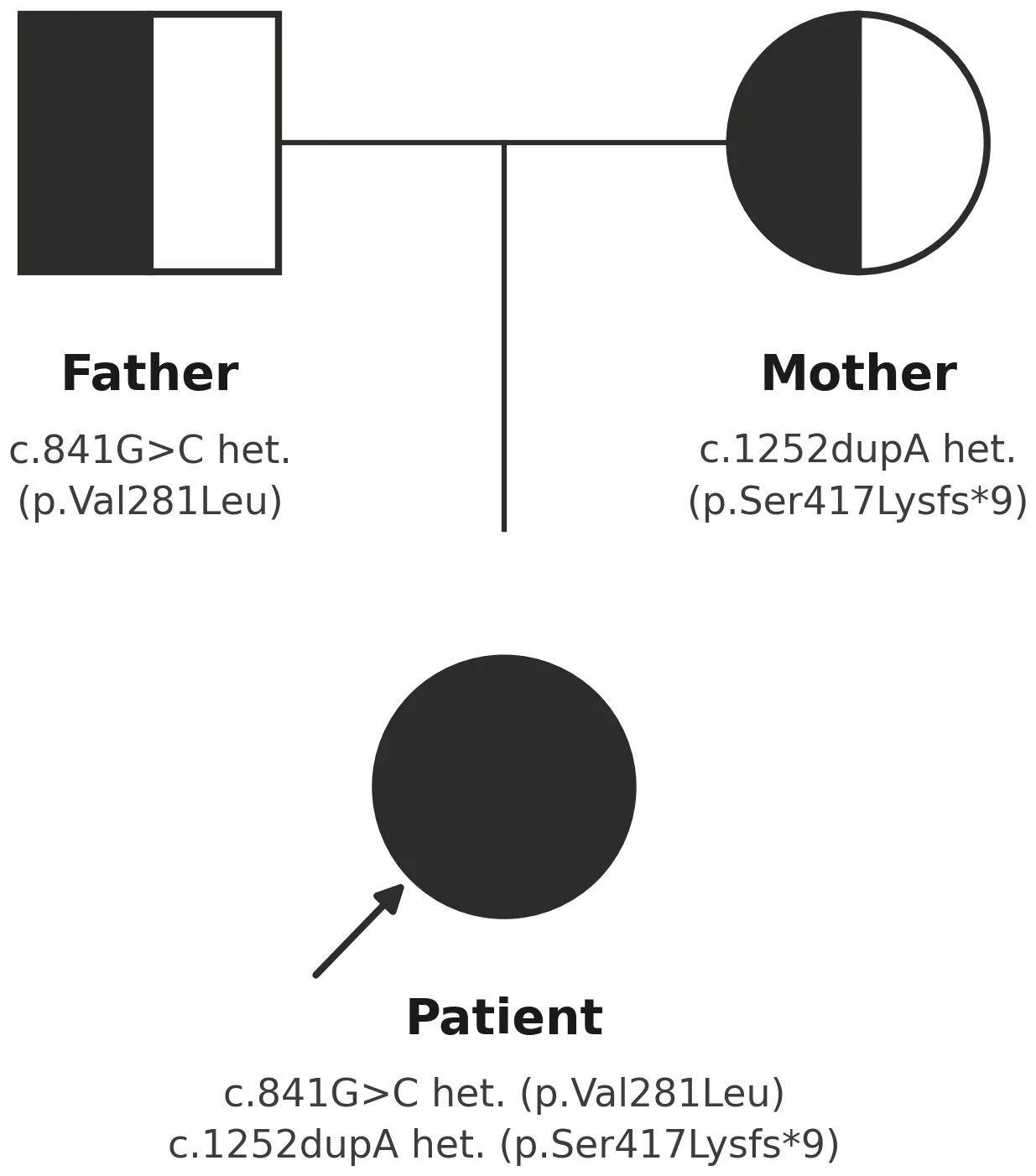
Standard pedigree showing the proband, and the unaffected father and the mother, consistent with autosomal recessive inheritance.

The patient, a 13-year-old female, was born to healthy, non-consanguineous parents. Perinatal course was uneventful, nevertheless since the first month of life, she experienced recurrent episodes of fever occurring every 1–2 months, without identifiable infectious triggers but associated with elevation of inflammatory markers. These episodes resolved with symptomatic treatment. Persistent mild microcytic and hypochromic anemia was also documented. She showed features suspicious for ectodermal dysplasia, including hair fragility, abnormal dental eruption, skin discoloration, and thin nails with longitudinal striations, according to Freire-Maia/Pinheiro nosology and its most recent international consensus update (15, 16), together with mild developmental delay, particularly involving the acquisition of upright posture and independent walking. At 5 years of age, she was hospitalized for severe bronchopneumonia and diarrhea, which responded to systemic antibiotics. Lung computed tomography (CT) showed multiple small nodules, bronchiectasis, atelectasis, air trapping, initial consolidations, and ground-glass opacities (**Figure 1**). Respiratory physiotherapy and antibiotic prophylaxis with azithromycin were started. Bone marrow examination both with Wright-Giemsa and Perls’ Prussian blue stain revealed erythroid hyperplasia with numerous ring sideroblasts, consistent with sideroblastic anemia. Consecutive serial immunological evaluation showed markedly reduced CD19^+^ B cells, reduced naïve B cells and switched memory B cells, low serum immunoglobulin G, A and M levels, and impaired responses to protein and polysaccharide vaccine antigens (**Table 1**). In addition, CD19^+^CD21^low^ B cells were markedly expanded. Anti-nuclear antibodies (ANA), extractable nuclear antigen antibodies (ENA), and C3 and C4 complement levels were within normal ranges. Intravenous immunoglobulin replacement therapy (IgRT) 0.4 g/kg every 3 weeks was started (switched subcutaneously at the age of 10 years), with good control on infectious episodes. Given the association of sideroblastic anemia, recurrent inflammatory episodes, immunodeficiency and immune dysregulation, an underlying genetic disorder was suspected. Next-generation sequencing (NGS), was performed at 7 years of age with a clinical exome sequencing approach with phenotype-driven *in silico* virtual panels focused on ectodermal dysplasias, immunoglobulin deficiency [suspected for predominantly antibody primary immunodeficiencies (PAD) of the common variable immunodeficiency (CVID) spectrum], recurrent fever syndrome/systemic autoinflammatory diseases (SAID), and microcytic anemia. It finally identified two biallelic *TRNT1* variants: c.1252dupA (p.Ser417Lysfs*9), a variant inherited from the healthy mother, and c.841G>C (p.Val281Leu), inherited from the healthy father and not previously reported (**Figure 2**). The c.1252dupA (p.Ser417Lysfs*9) variant is a frameshift duplication located in exon 8 that introduces a premature termination codon, resulting in truncation of the C-terminal portion of the TRNT1 protein. This variant has been previously reported in patients with TRNT1 deficiency and is classified as pathogenic in HGMD and ClinVar. It is extremely rare in the general population (gnomAD genomes homozygous allele count = 0). According to the American College of Medical Genetics and Genomics (ACMG) guidelines (17), this variant fulfills PVS1 (predicted loss-of-function in a gene where loss-of-function is an established disease mechanism), PM2 (absent/rare in population databases), and PP5 (ClinVar classifies this variant as Pathogenic citing 4 articles), supporting a pathogenic classification. The second variant, c.841G>C, is a missense change affecting a highly conserved amino acid within the catalytic domain of TRNT1. Although this variant has not been previously reported in affected individuals, it is absent or extremely rare in population databases (GnomAD allele frequency: 0). According to the ACMG guidelines, this variant fulfills PM2 (absent/rare in population databases), PM3 (For recessive disorders, detected in trans with a pathogenic variant) and PP4 (Patient’s phenotype or family history is highly specific for a disease with a single genetic etiology.), supporting a hot-VUS classification. Taken together these two variants confirmed the diagnosis of SIFD (17).

**Table 1:** Sequential immunological assessment of the patient. .

	Age of 5-year-old		Age of 6-year-old		Age of 8-year-old		Age of 10-year-old		Age of 12-year-old (pre-RTX)		Age of 13-year-old (6 months post-RTX)		Age of 14-year-old (2 years post-RTX)	
Laboratory analysis	Result	Reference range	Result	Reference range	Result	Reference range	Result	Reference range	Result	Reference range	Result	Reference range	Result	Reference range
IgG	**282 mg/dl^L^**	528–1959 mg/dl	**440 mg/dl^L^**	633–2016 mg/dl	**520 mg/dl^L^**	633–2016 mg/dl	603* mg/dl^L^	707–1919 mg/dl	905* mg/dl	640–1909 mg/dl	757* mg/dl	640–1909 mg/dl	716* mg/dl	640–1909 mg/dl
IgM	**20 mg/dl^L^**	49–292 mg/dl	**37 mg/dl^L^**	56–261 mg/dl	**14.2 mg/dl^L^**	56–261 mg/dl	20* mg/dl^L^	61–276 mg/dl	16* mg/dl^L^	59–297 mg/dl	8* mg/dl^L^	59–297 mg/dl	/	
IgA	**15 mg/dl^L^**	37–257 mg/dl	**13 mg/dl^L^**	41–315 mg/dl	**7.8 mg/dl^L^**	41–315 mg/dl	4* mg/dl^L^	60–270 mg/dl	4* mg/dl^L^	61–131 mg/dl	4* mg/dl^L^	61–131 mg/dl	/	
Lymphocytes	2.200/mm3	1.700-6.900/mm3	3.300/mm3	1.100-5.900/mm3	2.020/mm3	1.100-5.900/mm3	2.172/mm3	1.000-5.300/mm3	1.480/mm3	1.000-5.300/mm3	2.050/mm3	1.000-5.300/mm3	**900/mm3^L^**	1.000-5.300/mm3
CD3+T	**87%^H^**	43-76%	**85%^H^**	43-76%	**82%^H^**	43-76%	**91.1%^H^**	58-90%	**97%^H^**	52-78%	**91%^H^**	52-78%	**85%^H^**	52-78%
	1914/mm3	900-4500/mm3	2040/mm3	900-4500/mm3	1656/mm3	900-4500/mm3	1979/mm3	740-2731/mm3	1436/mm3	800-3500/mm3	1866/mm3	800-3500/mm3	**765/mm3^L^**	800-3500/mm3
CD4+T	24%	23-48%	46%	23-48%	**55%^H^**	23-48%	**71%^H^**	28-53%	**81%^H^**	25-48%	6**2%^H^**	25-48%	**51%^H^**	25-48%
	528/mm3	500-2400/mm3	1104/mm3	900-5500/mm3	1.111/mm3	900-5500/mm3	1542/mm3	379-1733/mm3	1199/mm3	400-2100/mm3	1271/mm3	400-2100/mm3	459/mm3	400-2100/mm3
CD8+T	23%	14-33%	**37%^H^**	14-33%	26%	14-33%	17.3%	12-32%	14%	9-35%	27%	9-35%	33%	9-35%
	506/mm3	300-1600/mm3	888/mm3	300-1600/mm3	525/mm3	300-1600/mm3	375/mm3	255-974/mm3	207/mm3	200-1200/mm3	554/mm3	200-1200/mm3	297/mm3	200-1200/mm3
CD19+B	**8%^L^**	14-44%	**4%L**	14-44%	**5%^L^**	14-44%	**3.4%^L^**	6-25%	**1%^L^**	8-24%	**0%^L^**	8-24%	**1%^L^**	8-24%
	**176/mm3^L^**	200-2100/mm3	**96/mm3^L^**	200-1600/mm3	**101/mm3^L^**	200-1600/mm3	**75/mm3^L^**	798-1220/mm3	15/mm3^L^	200-600/mm3	**0/mm3 ^L^**	200-600/mm3	**9/mm3 ^L^**	200-600/mm3
CD3-CD56+CD16+ NK	**3%^L^**	4-23%	7%	4-23%	4%	4-23%	5.2%	4-25%	**2%^L^**	6-27%	**2%^l^**	6-27%	9%	6-27%
	**66/mm3^L^**	100-1000/mm3	168/mm3	100-1000/mm3	**81/mm3^L^**	100-1000/mm3	113/mm3	79-918/mm3	**30/mm3^l^**	70-1200/mm3	144/mm3	70-1200/mm3	81/mm3	70-1200/mm3
CD3+ TCRapha/beta	**/**		**/**		**/**				**99%^H^**	88–98%	/		98%	88–98%
CD3+ TCR gamma/delta	**/**		**/**		**/**				**1%^L^**	2–12%	/		2%	2–12%
CD3+ TCRapha/beta CD4-CD8-	**/**		**/**		**/**				1%	<2.5%	/		1.1%	<2.5%
T-Naïve CD3+CD4+CD45RA+CD27+	**/**		**/**		**/**		28.6%	37-89%	**11%^L^**	37-89%	**/**		49%	37-89%
T-CM CD3+CD4+CD45RA-CD27+	**/**		**/**		**/**		19.9%	4-56%	27%	4-56%	/		20%	4-56%
T-EM CD3+CD4+CD45RA-CD27-	**/**		**/**		**/**		**50.1%^H^**	2-18%	**60%^H^**	2-18%	/		**27%^H^**	2-18%
T- EMRA CD3+CD4+CD45RA+CD27-	**/**		**/**		**/**		1.4%	1-15%	1%	1-15%	/		3%	1-15%
T-Naïve CD8+CD45RA+CD197+	**/**		**/**		**/**		53.6%	9-84%	28%	9-84%	/		31%	9-84%
T-CM CD8+CD45RA-CD197+	**/**		**/**		**/**		3.5%	1-34%	1%	1-34%	/		2%	1-34%
T-EM CD8+CD45RA-CD197-	**/**		**/**		**/**		**30.2%^H^**	2-19%	**54%^H^**	2-19%	/		**36%^H^**	2-19%
T-EMRA CD8+CD45RA+CD197-	**/**		**/**		**/**		12.7%	11-51%	16%	11-51%	/		31%	11-51%
B-Naïve CD19+CD27-IgM+IgD+	**88%^H^**	76.3-84.9%	70%	69.4-80.4%	73%	69.4-80.4%	**19.9%^L^**	69.4-80.4%	75.5%	75.2-86.7%	/		**94%^H^**	75.2-86.7%
B-Unswitched Memory CD19+CD27+IgM+IgD+	**3%^L^**	7.5-12.4%	**4%^L^**	7.5-12.4%	**21%^H^**	7.5-12.4%	**2.9%^L^**	7.5-12.4%	5.5%	4.6-10.2%	/		**3%^L^**	4.6-10.2%
B-Switched Memory CD19+CD27+IgM-IgD-	**2%^L^**	3.3-7.4%	**5%^L^**	5.2-12.1%	**3%^L^**	5.2-12.1%	**0.8%^L^**	5.2-12.1%	5.5%	3.3-9.6%	/		**0%^L^**	3.3-9.6%
B CD19+CD21^low CD38^low	**/**		**/**		**/**		**56.2%^L^**	0.7-3.5%	/		/		/	

*, values during IgRT; CM, central memory; EMRA, effector memory T cells re-expressing CD45RA; ^L^, low value; ^H^, high value; RTX, rituximab; TEM, effector memory T cells. H , increased levels; L, reduced levels. Bold values indicate results outside the reference range.

**Figure 3:**
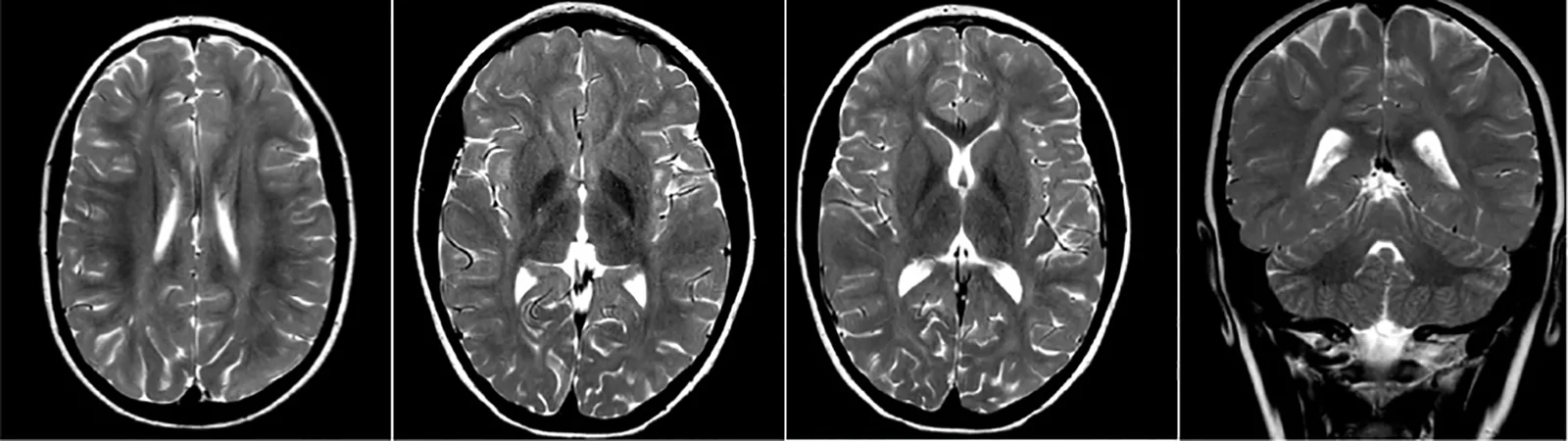
Brain magnetic resonance imaging findings. T2-weighted images showing diffuse white-matter hyperintensity involving the internal capsule and bilateral corona radiata, consistent with hypomyelinating leukodystrophy.

At 9 years of age, she developed night blindness with slow adaptation when moving from bright to dark environments. Ophthalmological evaluation, including electroretinogram (ERG), showed features of retinal dystrophy. At 10 years of age, she was hospitalized for a generalized tonic-clonic seizure. Electroencephalogram (EEG) demonstrated a non-specific spike-and-wave pattern, while brain magnetic resonance imaging (MRI) showed findings suggestive of bi-hemispheric supratentorial hypomyelinating leukodystrophy (**Figure 3**). After this episode, the patient reported persistent generalized tremor and balance impairment, with a non-conclusive EEG.

At 12 years of age, she was hospitalized for severe thrombocytopenia, with a platelet count of 1,000/mm³. She was treated with two courses of high-dose intravenous immunoglobulin (IVIG; 0.8 g/kg), followed by high-dose oral corticosteroids because of poor cytopenia responsiveness. Extensive autoimmune screening, including ANA, ENA, and anti-double-stranded DNA antibodies, as well as infectious investigations, were largely unrevealing, except for low-level detection of human herpesvirus 7 (HHV-7) and Epstein–Barr virus (EBV) DNA. During this episode, cytokine abnormalities were detected (**Table 2**). Six months after discharge, she was readmitted because of severe autoimmune pancytopenia and splenomegaly, with a splenic length of 20 cm on abdominal ultrasound (US) imaging. Due to a slight increase in B-cell and subset counts (**Table 1**), despite still being below normal age-matched range, treatment with rituximab (RTX) was started with four weekly doses. RTX finally led to progressive improvement in hemoglobin, platelet count, and white blood cell count, together with a reduction in splenic size to 14 cm on the US examination performed after four months from the last rituximab administration. No additional RTX administration was required. Conversely, lung CT revealed progression of the previously reported ground-glass opacities, with microbiological investigations, including bronchoalveolar lavage, resulting negative.

**Table 2:** Cytokine analysis of our patient during the acute phase of immune thrombocytopenia.

Cytokine	Value	Reference range
TGF-β	↑ **11202** pg/ml	1414-4641 pg/ml
IL-6	↑ **34** pg/ml	0-13.90 pg/ml
IL-1β	0.1 pg/ml	0-3.90 pg/ml
IL-10	↑ **78** pg/ml	0-7.80 pg/ml
IL-2	9 pg/ml	0-31.20 pg/ml
TNF-α	↑ **74** pg/ml	0-15.60 pg/ml
IL-8	↑ **104** pg/ml	0-31.20 pg/ml

↑ , increased level. Bold values indicate results outside the reference range.

**Figure 4:**
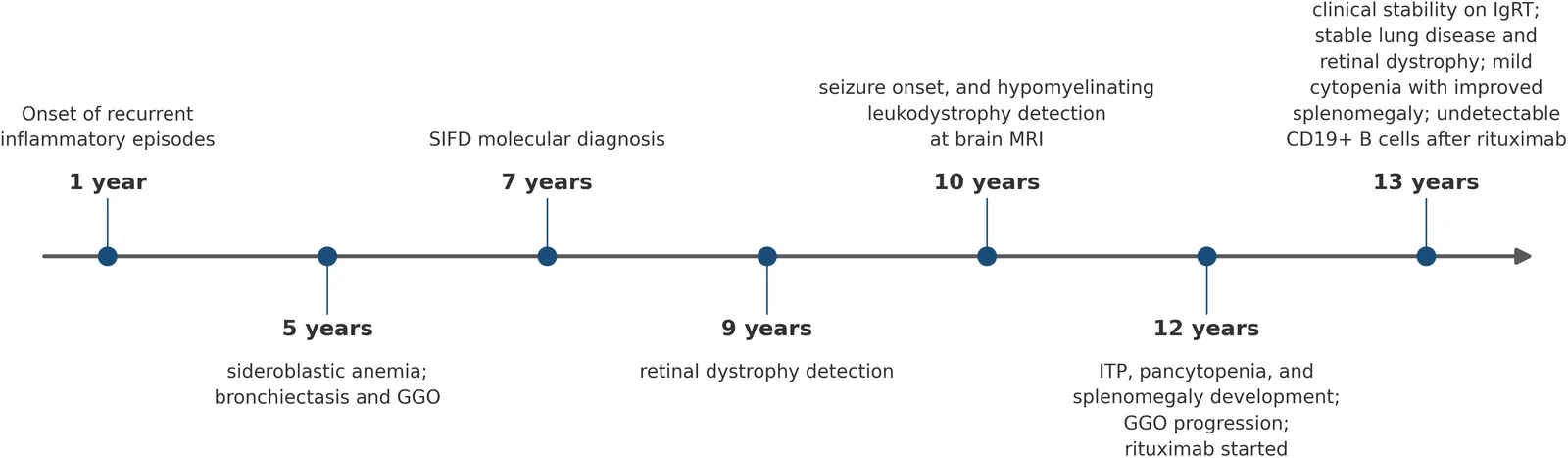
Timeline of the main disease manifestations, diagnostic steps, and therapeutic interventions during the patient’s follow-up. GGO, ground glass opacities; ITP, immune thrombocytopenia; IgRT, immunoglobulin replacement therapy.

At the last follow-up, the patient continues to receive IgRT every 3 weeks and remains under neurological, ophthalmological, and pulmonary monitoring. She has persistent mild cytopenia and splenomegaly, stable lung disease, and fewer fever episodes, occurring approximately 2–3 times per year. Cardiac and renal ultrasound examinations showed no abnormalities. No additional signs of neurodevelopmental impairment were observed, except for mild ataxia. Sequential immunophenotyping after rituximab showed undetectable CD19+B-cells. **Figure 4** represents a timeline showing the main disease manifestations, diagnostic steps, and therapeutic interventions during the patient’s follow-up. 

## 3 Discussion

Here, we report a pediatric patient with SIFD presenting with a complex and progressive phenotype characterized by sideroblastic anemia, recurrent inflammatory episodes, B-cell immunodeficiency, severe immune dysregulation, ectodermal abnormalities, neurodevelopmental involvement, hypomyelinating leukodystrophy, retinal dystrophy, chronic lung disease, cytopenias, and splenomegaly. This case further expands the clinical, immunological, and genetic spectrum of TRNT1-related disease (18, 19).

*TRNT1* is ubiquitously expressed and is essential for both mitochondrial and cytosolic protein synthesis. Loss-of-function variants lead to defective tRNA maturation, impaired oxidative phosphorylation, accumulation of misfolded proteins, cellular stress responses, and activation of inflammatory pathways (1, 3). Mitochondrial dysfunction is thought to play a central role in disease pathogenesis, particularly in high-energy-demand tissues such as the bone marrow, nervous system, and immune cells (4). Moreover, TRNT1 has been shown to contribute to pathogen-specific mitochondrial reprogramming in macrophages, thereby influencing inflammatory responses (1). As mitochondrial stress and inflammatory pathway activation underlie the febrile episodes of SIFD, it is important to distinguish these autoinflammatory fevers from the non-inflammatory, thermoregulation-related hyperthermic episodes (so-called “sterile fevers”) described in patients with classic hypohidrotic/anhidrotic ectodermal dysplasia (15, 16).

Since its initial description, fewer than 100 cases of SIFD have been reported worldwide (5). The phenotype ranges from severe early-onset multisystem disease to milder forms with predominant hematological involvement. However, a clear genotype–phenotype correlation remains difficult to establish. The heterogeneous clinical manifestations of SIFD are thought to reflect altered tRNA maturation and abundance, resulting in impaired translation of distinct proteins and variable tissue involvement (20, 21).

Over the last few years, increasing evidence has highlighted the broad clinical heterogeneity of SIFD, as well as differences in therapeutic management and outcomes (7–14). Common manifestations include sideroblastic anemia, recurrent fever, immunodeficiency, developmental delay, failure to thrive, ophthalmological abnormalities, sensorineural hearing loss, and cardiac or renal involvement, including hypertrophic or dilated cardiomyopathy, Fanconi syndrome, and nephrocalcinosis (20–22). Additional rare manifestations, such as mitochondrial myopathy, spontaneous bilateral osteonecrosis of the femoral heads, growth hormone deficiency, hypoglycemia, primary hypogonadism, and delayed-onset milder phenotypes, have also been reported (12–14, 23). Sideroblastic anemia remains one of the most frequent and earliest manifestations and may be transfusion-dependent (18).

Neurological manifestations are increasingly recognized in SIFD and likely reflect the vulnerability of neuronal and glial cells to mitochondrial dysfunction. Our patient developed persistent tremor and gait instability, with MRI findings consistent with supratentorial hypomyelinating leukodystrophy. Previous reports have described neurological involvement, including ataxia, cerebellar abnormalities, cerebral atrophy of variable severity, delayed white-matter myelination, abnormal enhancement of the external capsule and thalamus, communicating hydrocephalus, and reduced cerebellar perfusion (1, 6, 7, 11, 18, 23). The presence of ectodermal dysplasia affecting hair, teeth, skin, and nails further supports a broader role of TRNT1 in epithelial tissue development and maintenance, consistent with previous reports of ectodermal involvement in SIFD (19).

Immunodeficiency with immune dysregulation represents a key feature of SIFD and may manifest as recurrent infections, autoimmunity, autoinflammatory episodes, cytopenias, and lymphoproliferation (18). Immunological abnormalities reported in SIFD include hypogammaglobulinemia, impaired vaccine responses, reduced circulating CD19^+^ B cells, reduced memory B cells, and decreased switched memory B cells, supporting an early defect in B-cell maturation (21–24). Of note, our patient showed a marked expansion of CD19^+^CD21^low^ B cells, a B-cell subset frequently expanded in chronic inflammatory and autoimmune conditions (25). This finding may reflect the severe immune dysregulation phenotype observed in this case, including refractory cytopenias, splenomegaly, and chronic lung involvement (26). Consistently, T-cell analysis revealed increased proportions of CD4^+^ T cells and effector memory subsets, suggesting chronic immune activation and skewing toward terminal differentiation, as commonly observed in immune dysregulation syndromes. Severe reductions in T-cell receptor excision circles and kappa-deleting recombination excision circles have also been reported in SIFD, further supporting the combined nature of the immune defect (18, 19). These findings highlight the need for close immunological monitoring and early initiation of immunoglobulin replacement therapy.

Management of SIFD remains challenging and is largely supportive. Red blood cell transfusions and iron chelation may be required in patients with severe or transfusion-dependent anemia, while immunoglobulin replacement therapy can reduce infectious complications in patients with antibody deficiency. In addition to corticosteroids, several immunomodulatory and biologic treatments have been used to control autoinflammatory and immune dysregulation manifestations, including colchicine, methotrexate, azathioprine, thalidomide, anti-TNF agents, and IL-1 blockade (6, 9–12, 18). These treatments have shown clinical benefit in selected patients, particularly for febrile episodes and systemic, articular, gastrointestinal, and neurological inflammatory manifestations (6, 9–12, 18).

Cytokine abnormalities have also been described in SIFD, including increased levels of IL-1β, soluble IL-2 receptor, IL-6, IL-8, IL-18, TNF-α, IL-10, and interferon-α–induced proteins, either during febrile episodes or in clinically active disease (6, 11). Consistent with these observations, our patient showed cytokine abnormalities during the immune thrombocytopenia episode. These findings support the relevance of inflammatory pathway activation in SIFD and may help guide targeted therapeutic approaches in selected cases.

Hematopoietic stem cell transplantation has been reported in a limited number of patients with severe SIFD and remains the only potentially curative option (1, 22). However, outcomes are variable, ranging from long-term survival with resolution of fever, normalization of hematological and immunological parameters, and improved growth and development, to early mortality due to graft rejection, severe infections, neurological complications, and multiorgan failure (1, 2, 4, 5, 11, 22). Therefore, HSCT should be considered carefully, balancing disease severity, donor availability, organ involvement, and transplant-related risks. Our patient currently lacks a suitable donor; nevertheless, despite her complex and progressive phenotype, she has achieved partial disease control with supportive and immunomodulatory treatment. Detailed information on the conditioning regimens used in previously reported SIFD patients who underwent HSCT remains limited (e.g. reduced-toxicity conditioning with fludarabine, treosulfan, thiotepa, and alemtuzumab), and the small number of cases described to date illustrates substantial heterogeneity in both transplant approach and outcome (1, 2, 4, 5, 11, 22). Regimen choice alone does not fully account for the variability observed, and age at transplant, pre-existing organ involvement, and donor source likely also contribute substantially to prognosis. Given the progressive multisystem organ involvement frequently observed in SIFD, careful pre-transplant assessment of organ function and individualized weighing of transplant-related risks against those of disease progression appear essential, although the current evidence base remains too limited to support a specific conditioning strategy.

The use of rituximab in our patient was justified by the presence of refractory cytopenias, splenomegaly, and lymphoproliferative/immune dysregulation features. Rituximab is widely used for autoimmune cytopenias and lymphoproliferative manifestations in several immune dysregulation disorders, including CVID-like conditions (25). In this case, rituximab led to improvement in blood counts and reduction of splenic size, suggesting that B-cell–directed therapy may be considered in selected SIFD patients with prominent autoimmune or lymphoproliferative manifestations.

Finally, we report a novel *TRNT1* variant [c.841G>C (p.Val281Leu)], further expanding the mutational spectrum of SIFD. The association of a frameshift variant with a missense variant may contribute to the severe multisystem involvement observed in our patient, although definitive genotype–phenotype correlations in SIFD remain limited (6, 20). Although one of the two variants remains classified as a hot-VUS, no additional pathogenic, likely pathogenic, or clinically relevant variants were identified through either phenotype-driven or unbiased clinical exome analysis. Moreover, the patient’s phenotype closely matches that reported in individuals with biallelic *TRNT1* variants. Taken together, the absence of alternative molecular findings and the strong genotype–phenotype correlation support *TRNT1* as the most likely molecular diagnosis in this patient. Overall, this case supports the role of *TRNT1* in immune homeostasis and highlights the importance of considering SIFD in children with sideroblastic anemia, recurrent unexplained inflammation, progressive B-cell immunodeficiency, cytopenias, and multisystem immune dysregulation.

The single-case design, which precludes definitive genotype–phenotype correlation, and the lack of functional validation of the novel variant may represent the main limitations. Therefore, larger, prospectively followed cohort studies are needed to generalize these findings to the broader SIFD population.

## 4 Conclusion

SIFD is a complex multisystem disorder with marked clinical, immunological, and genetic heterogeneity. This case expands the spectrum of *TRNT1*-related disease and highlights that immune dysregulation, including progressive B-cell immunodeficiency, cytopenias, splenomegaly, and chronic lung involvement, may represent a major component of the clinical course. Comprehensive immunological assessment and early genetic diagnosis are essential to guide monitoring, therapeutic decisions, and prognostic stratification. Further studies are needed to better define genotype–phenotype correlations and to refine targeted therapeutic strategies for patients with SIFD.

## 5 Patient perspective

Written informed consent was obtained from the patient’s legal guardians (the parents), as well as from the subject involved in the study. Additionally, the legal guardian took part in the development of the case report, which they reviewed before its completion. Their feedback and suggestions have been incorporated into the final version.

Ethical approval was not required for this study because it describes a single clinical case and did not involve experimental procedures, interventional research, or the collection of additional data beyond routine clinical care.

## Data Availability

The original contributions presented in the study are included in the article/supplementary material. Further inquiries can be directed to the corresponding author.

## References

[1] Wiseman DH May A Jolles S Connor P Powell C Heeney MM . A novel syndrome of congenital sideroblastic anemia, B-cell immunodeficiency, periodic fevers, and developmental delay (SIFD). Blood. (2013) 122:112–23. doi: 10.1182/blood-2012-08-439083 23553769 PMC3761334

[2] Chakraborty PK Schmitz-Abe K Kennedy EK Mamady H Naas T Durie D . Mutations in TRNT1 cause congenital sideroblastic anemia with immunodeficiency, fevers, and developmental delay (SIFD). Blood. (2014) 124:2867–71. doi: 10.1182/blood-2014-08-591370 25193871 PMC4215314

[3] Nagaike T Suzuki T Tomari Y Takemoto-Hori C Negayama F Watanabe K . Identification and characterization of mammalian mitochondrial tRNA nucleotidyltransferases. J Biol Chem. (2001) 276:40041–9. doi: 10.1074/jbc.M106202200 11504732

[4] Giannelou A Wang H Zhou Q Park YH Abu-Asab MS Ylaya K . Aberrant tRNA processing causes an autoinflammatory syndrome responsive to TNF inhibitors. Ann Rheum Dis. (2018) 77:612–9. doi: 10.1136/annrheumdis-2017-212401 29358286 PMC5890629

[5] Wedatilake Y Niazi R Fassone E Powell CA Pearce S Plagnol V . TRNT1 deficiency: clinical, biochemical and molecular genetic features. Orphanet J Rare Dis. (2016) 11:90. doi: 10.1186/s13023-016-0477-0 27370603 PMC4930608

[6] Maccora I Ramanan AV Wiseman D Marrani E Mastrolia MV Simonini G . Clinical and therapeutic aspects of sideroblastic anaemia with B-cell immunodefi-ciency, periodic fever and developmental delay (SIFD) syndrome: a systematic review. J ClinImmunol.. (2023) 43:1–30. doi: 10.1007/s10875-022-01343-0 35984545 PMC9840570

[7] Wang J Deng Q He X Chen D Hang S Gao Y . Two cases of sideroblastic anemia with B-cell immunodeficiency, periodic fevers, and developmental delay (SIFD) syndrome in Chinese Han children caused by novel compound heterozygous variants of the TRNT1 gene. Clin Chim Acta. (2021) 521:244–50. doi: 10.1016/j.cca.2021.07.019 34310935

[8] Wang J He X Chen D Hang S Gao Y Li X . A case of SIFD syndrome caused by novel compound heterozygous variants of TRNT1 gene. Zhonghua Yi Xue Yi Chuan Xue Za Zhi.. (2021) 38:977–80. doi: 10.3760/cma.j.cn511374-20200716-00517 34625936

[9] Kisla Ekinci RM Zararsiz A Urel Demir G Anlas O . A rare autoinflammatory disorder in a pediatric patient with favorable response to etanercept: Sideroblastic anemia with B cell immunodeficiency, periodic fevers, and developmental delay syndrome. Pediatr Allergy Immunol Pulmonology. (2022) 35:129–32. doi: 10.1089/ped.2022.0090 36121781

[10] Li Y Deng M Han T Mo W Mao H . Thalidomide as an effective treatment in sideroblastic anemia, immunodeficiency, periodic fevers, and developmental delay (SIFD). J ClinImmunol.. (2023) 43:780–93. doi: 10.1007/s10875-023-01441-7 36729249 PMC9893968

[11] Chen X Fu F Mo X Cheng S Zeng H . Case report: Sideroblastic anemia with B-cell immunodeficiency, periodic fevers, and developmental delay: Three cases and a literature review. Front Pediatr. (2023) 11:1001222. doi: 10.3389/fped.2023.1001222 36937953 PMC10017860

[12] Cai G Jayaraman D . Spontaneous, simultaneous bilateral osteonecrosis of the femoral heads in a patient with sideroblastic anaemia with B-cell immunodeficiency, periodic fever and developmental delay syndrome. BMJ Case Rep CP. (2023) 16:e254175. doi: 10.1136/bcr-2022-254175 37130647 PMC10163426

[13] Bravo García-Morato M Padilla-Merlano B Matas Pérez E Valdivieso Shephard JL Robles Marhuenda Á . Hypomorphic variant in TRNT1 induces a milder autoinflammatory disease with congenital cataracts and impaired sexual development. Rheumatol (Oxford). (2022) 61:e114–6. doi: 10.1093/rheumatology/keab903 34864912

[14] Odom J Amin H Gijavanekar C Elsea SH Kralik S Chinen J . A phenotypic expansion of TRNT1 associated sideroblastic anemia with immunodeficiency, fevers, and developmental delay. Am J Med Genet A. (2022) 188:259–68. doi: 10.1002/ajmg.a.62482 34510712

[15] Pinheiro M Freire-Maia N . Ectodermal dysplasias: A clinical classification and a causal review. Am J Med Genet. (1994) 53:153–62. doi: 10.1002/ajmg.1320530207 7856640

[16] Wright JT Fete M Schneider H Zinser M Koster MI Clarke AJ . Ectodermal dysplasias: Classification and organization by phenotype, genotype and molecular pathway. Am J Med Genet A. (2019) 179:442–7. doi: 10.1002/ajmg.a.61045 30703280 PMC6421567

[17] Richards S Aziz N Bale S Bick D Das S Gastier-Foster J . Standards and guidelines for the interpretation of sequence variants: a joint consensus recommendation of the American College of Medical Genetics and Genomics and the Association for Molecular Pathology. Genet Med. (2015) 17:405–24. doi: 10.1038/gim.2015.30 25741868 PMC4544753

[18] Rigante D Stellacci E Leoni C Onesimo R Radio FC Pizzi S . Biallelic TRNT1 variants in a child with B cell immunodeficiency, periodic fever and developmental delay without sideroblastic anemia (SIFD variant). Immunol Lett. (2020) 225:64–5. doi: 10.1016/j.imlet.2020.06.012 32592741

[19] Mendonca LO Prado AI Costa IMC Bandeira M Dyer R Barros SF . Case Report: Expanding Clinical, Immunological and Genetic Findings in Sideroblastic Anemia With Immunodeficiency, Fevers and Development Delay (SIFD) Syndrome. Front Immunol. (2021) 12:586320. doi: 10.3389/fimmu.2021.586320 33936027 PMC8079983

[20] Fouquet C Le Rouzic MA Leblanc T Fouyssac F Leverger G Hessissen L . Genotype/phenotype correlations of childhood-onset congenital sideroblastic anaemia in a European cohort. Br J Haematol. (2019) 187:530–42. 10.1111/bjh.16100 31338833

[21] Kumaki E Tanaka K Imai K Aoki-Nogami Y Ishiguro A Okada S . Atypical SIFD with novel TRNT1 mutations: a case study on the pathogenesis of B-cell deficiency. Int J Hematol. (2019) 109:382–9. doi: 10.1007/s12185-019-02614-0 30758723

[22] Barton C Kausar S Kerr D Bitetti S Wynn R . SIFD as a novel cause of severe fetal hydrops and neonatal anaemia with iron loading and marked extramedullary haemopoiesis. J Clin Pathol. (2018) 71:275–8. doi: 10.1136/jclinpath-2017-204698 29055896 PMC5868532

[23] Wei CJ Liu YD Yang YL Wu Y Liu JY Chang XZ . Case report: Muscle involvement in a Chinese patient with TRNT1-related disorder. Front Pediatr. (2023) 11:1160107. doi: 10.3389/fped.2023.1160107 37215601 PMC10196124

[24] Poli MC Aksentijevich I Bousfiha AA Cunningham-Rundles C Hambleton S Klein C . Human inborn errors of immunity: 2024 update on the classification from the International Union of Immunological Societies Expert Committee. J Hum Immun. (2025) 1:e20250003. doi: 10.70962/jhi.20250003 41608114 PMC12829761

[25] Gjertsson I McGrath S Grimstad K Jonsson CA Camponeschi A Thorarinsdottir K . A close-up on the expanding landscape of CD21-/low B cells in humans. Clin Exp Immunol. (2022) 210:217–29. doi: 10.1093/cei/uxac103 36380692 PMC9985162

[26] Greinix HT Kuzmina Z Weigl R Körmoczi U Rottal A Wolff D . CD19+CD21low B cells and CD4+CD45RA+CD31+ T cells correlate with first diagnosis of chronic graft-versus-host disease. Biol Blood Marrow Transplant.. (2015) 21:250–8. doi: 10.1016/j.bbmt.2014.11.010 25460358

